# Affective Beliefs Influence the Experience of Eating Meat

**DOI:** 10.1371/journal.pone.0160424

**Published:** 2016-08-24

**Authors:** Eric C. Anderson, Lisa Feldman Barrett

**Affiliations:** 1 Center for Applied Brain and Cognitive Science, Tufts University, Medford, Massachusetts, United States of America; 2 Department of Psychology, Northeastern University, Boston, Massachusetts, United States of America; 3 Massachusetts General Hospital and Harvard Medical School, Boston, Massachusetts, United States of America; Universidade do Porto Instituto de Biologia Molecular e Celular, PORTUGAL

## Abstract

People believe they experience the world objectively, but research continually demonstrates that beliefs influence perception. Emerging research indicates that beliefs influence the experience of eating. In three studies, we test whether beliefs about how animals are raised can influence the experience of eating meat. Samples of meat were paired with descriptions of animals raised on factory farms or raised on humane farms. Importantly, the meat samples in both conditions were identical. However, participants experienced the samples differently: meat paired with factory farm descriptions looked, smelled, and tasted less pleasant. Even basic properties of flavor were influenced: factory farmed samples tasted more salty and greasy. Finally, actual behavior was influenced: participants consumed less when samples were paired with factory farm descriptions. These findings demonstrate that the experience of eating is not determined solely by physical properties of stimuli—beliefs also shape experience.

## Introduction

Every day, we must decide what’s for dinner. In our evolutionary past, humans had to avoid poisonous foods, making eating decisions a matter of life and death. In the 21st century, eating choices still matter: diet plays a role in heart disease [1], obesity [2], diabetes [3], and stroke [4]. One factor that drives food choice is the experience of eating: we eat ice cream on a hot day because we enjoy the experience of cold, creamy sweetness. While part of the experience of eating is due to the physical properties of food, incoming gustatory sensory signals are interpreted and experienced differently depending on what the brain believes, is expecting, or is predicting (for reviews see [5,6]). Beliefs about food are particularly important to understand because they are shaped by culture, education, and advertising. In this paper, we explore whether beliefs about animal suffering can influence the experience of eating meat.

Beliefs influence how people evaluate food. Wine tastes better when people believe it is expensive, compared to when they believe it is inexpensive—even when the two wine samples are actually identical [7]. Savory broth is experienced as more pleasant when labeled as a ‘rich and delicious taste’ compared to when labeled as ‘monosodium glutamate’ [8]. People enjoy salmon flavored ice cream if they believe it is savory frozen mousse, but they dislike the same substance if they are expecting a traditional sweet ice cream [9]. Moreover, beliefs have a different effect if presented before or after consumption, suggesting beliefs change the actual consumption experience—not just how participants report the experience [10].

Beliefs about *how* food is produced also influence the experience of eating. For instance, organic labels can influence the pleasantness and flavor of food [11,12]. Coffee labeled “eco-friendly” tastes better to people than identical unlabeled coffee [13]. Believing food was produced ethically (e.g. fair trade or local) increases the enjoyment of food [12]. These examples also demonstrate that *how* food is produced is an ethical issue for some people, which is increasingly important for purchasing decisions and can influence experience (for discussion see [12]).

Beliefs about the production of meat are particularly impactful. For instance, some have argued that animals suffer greatly in current industrial farming operations [14,15], and this motivates some people to avoid eating meat entirely (i.e. vegetarians, for review see [16]; though vegetarians avoid meat for other reasons as well, see [17]). Even many omnivores agree that raising animals for meat causes suffering, which results in omnivores experiencing the 'Meat Paradox': meat is delicious but causes suffering [18,19]. One way to diminish this dilemma is through reducing animal suffering by improving the conditions in which animals are raised. In fact, businesses and marketers have started to capitalize on omnivores' concern for animal welfare by certifying some meat as humanely raised (examples include the Global Animal Partnership in the United States [20] and the Beter Leven labels in the Netherlands [21]). While some consumers value animal welfare and are willing to pay more for humanely raised products, no research has examined whether beliefs about how animals are raised can influence the actual experience of consuming animal products.

Our goal was to test whether beliefs about how animals were raised (whether they suffered) would influence the experience of eating meat. According to a grounded cognition perspective, beliefs are instances of conceptual knowledge that include affective and sensory neural representations (for a discussion, see [22]). Therefore, beliefs that meat came from animals that suffered would be represented, in part, in regions of the brain that are associated with embodied simulation of animals’ experience (mirroring; for review see [23]). Animal suffering is typically affectively negative for people: pictures of animal suffering triggers negative affective feelings [24]. Therefore, beliefs that animals suffered in the production of a meat would be affectively negative. We hypothesized that these negative affective beliefs (that animals suffered) would reduce the pleasantness of eating meat. We focused on the pleasantness of the eating experience (also referred to as the ‘hedonic’ experience) because it is an important consideration in eating [25], and product consumption more broadly [26]. In Study 1, we tested the hypothesis that beliefs influence eating by manipulating people's beliefs about two identical meat samples: people were led to believe one sample was raised on a 'factory farm' (negative belief) while the other was 'humanely raised' (positive belief). In Study 2, we used a control condition to test whether negative or positive beliefs were more impactful. Finally, in Study 3, we tested an even more compelling possibility: can beliefs influence basic *sensory* properties of flavor, such as perceived saltiness or sweetness? In all three studies, participants first read the descriptions, then consumed real meat samples, and finally reported on their eating experience. To make sure any observed differences were not due to physical differences between the samples, all samples within each study were always identical.

## Study 1

### Methods

#### Participants

146 Northeastern University undergraduate students participated in Study 1. An initial sample size of n = 150 was set a priori based on previous studies exploring similar questions [11]. Data collection proceeded until the predetermined sample size was reached or until the semester concluded, which constrained our ability to collect data. 29 participants did not follow instructions (did not carefully read the descriptions) so were removed from analysis (see below for details; the pattern of findings does not change with all participants included). The remaining 117 participants who were analyzed included 72 females (61.5%) and ranged in age from 17–23 years old (*M* = 18.74, *SD* = 1.05). Participants were recruited through introductory psychology classes and received course credit for participation. All studies and consent procedures were approved by Northeastern University's Institutional Review Board. Participants gave written, informed consent before participating (parents or guardians also gave written, informed consent if participants were under 18 years old).

#### Materials

To manipulate participants' beliefs about meat, two labels were prepared that described two different farms on which cows were raised (S1 Table). The 'humane farm' label described a farm where animals were raised relatively humanely (i.e. animals grazed outdoors). The 'factory farm' label described a farm where animals were raised in relatively inhumane conditions (i.e. animals confined to indoor pens). Each participant was exposed to both labels in randomized order.

Two identical samples were prepared. Each consisted of two grams beef jerky cut into ten pieces. Each was placed in the center of a white plate for participants to sample. To control for the physical properties of the sample, the meat in all conditions was the same organic beef jerky. This was important because the farm environment can influences the physical properties of meat. For instance, grass-fed animals have increased beta-carotene, vitamin E, folic acid, and Omega 3 fatty acid as well as reduced fat [27]. During the experiment, each participant consumed two identical samples, but each sample was paired with a different description to manipulate the participants’ beliefs.

#### Procedure

After arriving in the lab and consenting, participants completed two trials. For each trial, participants were first instructed to cleanse their palate by sipping a glass of water. Next, participants read one of the descriptions of beef jerky (S1 Table), consumed a sample, and rated the sample (described in more detail below). Then participants completed a second trial with the other description and another (identical) sample. Therefore each participant completed two trials (tasted two samples paired with two different descriptions; order randomized). In reality, the actual samples were the same beef jerky product on both trials. Participants also completed two filler taste tasks in which they sampled two raisin products using a similar procedure.

After reading the description, participants reported their experience of the sample using 100-point slider scales on a computer. First participants were instructed to look at the sample and rate its appearance (from 0 = ‘very unappealing’ to 100 = ‘very appealing’). Next they smelled the sample and rated its smell (from 0 = ‘very unappealing’ to 100 = ‘very appealing’). Then participants tasted the sample and rated its taste (from 0 = ‘not very good’ to 100 = ‘very good’). They were also asked to report overall how much they enjoyed the sample (from 0 = ‘did not enjoy’ to 100 = ‘enjoyed very much’). Next, participants reported how much they would be willing to pay for a 6 ounce package of the sample (from $0 to $20), and how likely they would be to eat the sample again (from 0 = ‘would never eat it again’ to 100 = ‘would definitely eat it again’). Finally, to quantify an implicit measure of liking, we measured how much of the sample participants consumed by measuring the weight of the samples before and after the sampling procedure. Participants were free to eat as much of the samples as they wanted so the amount consumed could vary from zero to two grams.

To check whether participants were attending to the descriptions of the samples, participants were immediately asked to recall as much of each description as possible after they completed the sampling procedure. 29 participants did not recall any parts of the descriptions suggesting they did not read them. These participants were removed from analysis, but including them did not change the pattern of findings. Finally, participants were instructed that they could chose to not answer any questions, so some analyses contain fewer observations.

### Results

**Fig 1:**
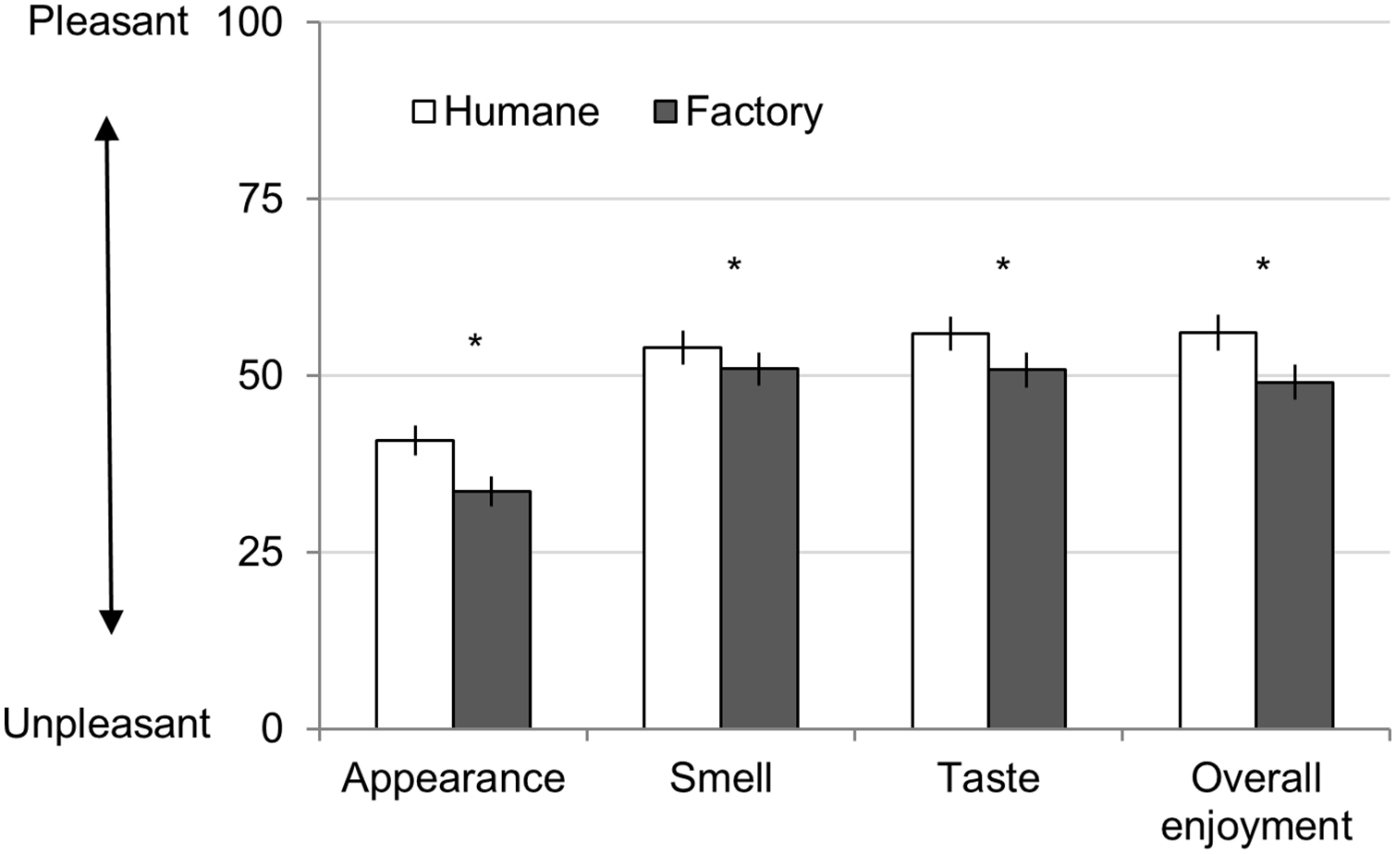
Study 1: Ratings of Beef Jerky Sample. Error bars represent standard errors, and asterisks represent significance (* *p* < 0.05). All ratings made on 100-point slider scale.

**Fig 2:**
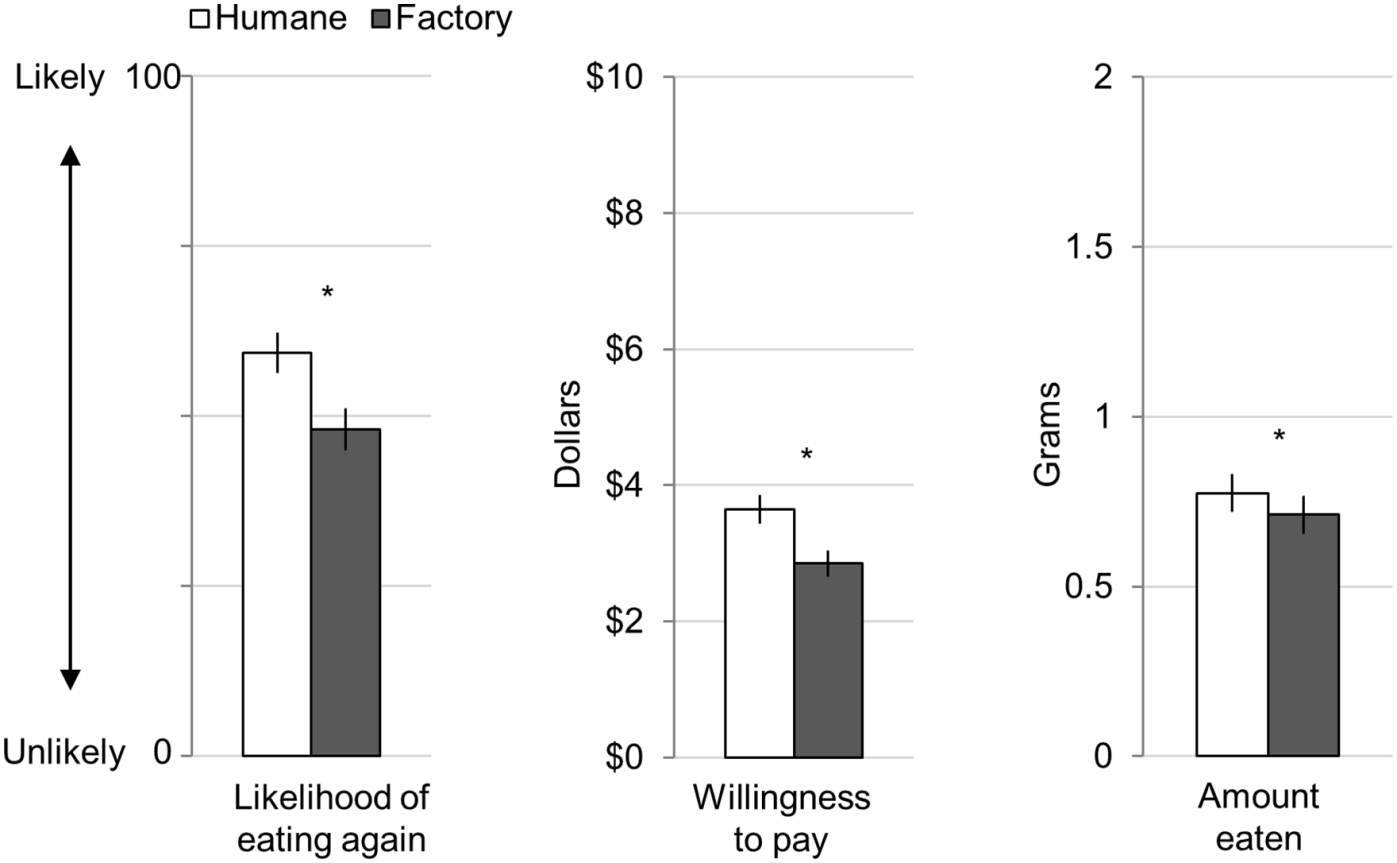
Study 1: Behavioral Measures for Beef Jerky Sample. Error bars represent standard errors, and asterisks represent significance (* *p* < 0.05).

To test whether beliefs influenced the experience of eating, we first conducted a MANOVA with description (humane farm vs. factory farm) as a repeated measure and participants’ ratings (appearance, smell, taste, overall experience, willingness to pay, likelihood of eating again, and amount eaten) as the dependent variables. The MANOVA was significant, Wilks' Lambda = 0.59, *F*(7,93) = 9.08, *p* < 0.001, η^2^_p_ = 0.406. To understand how the dependent variables were influenced, we ran a series of univariate tests (paired two-tailed t-tests) with the description (humane farm vs. factory farm) as the independent (within participant) variable. Supporting our hypothesis, participants reported the factory farm meat sample was less pleasant along all of the consumption dimensions we measured (compared to the humane farm sample; see Fig 1; see S2 Table for means, standard error (SE), and 95% confidence interval (95% CI)). Factory farm samples (compared to humane farm) looked less appealing, *t*(110) = 5.09, *p* < 0.001, η^2^_p_ = 0.19; smelled less appealing, *t*(111) = 2.47, *p* < 0.016, η^2^_p_ = 0.052; tasted worse, *t*(113) = 3.10, *p* < 0.003, η^2^_p_ = 0.078; and the overall consumption experience was less enjoyable, *t*(114) = 4.17, *p* < 0.001, η^2^_p_ = 0.132. Additionally, participants were willing to pay 21.82% less for the factory farmed jerky, *t*(111) = 6.64, *p* < 0.001, η^2^_p_ = 0.284, and participants reported being less likely to eat it again, *t*(110) = 5.86, *p* < 0.001, η^2^_p_ = 0.238. Importantly, participants also consumed 8.18% less of the factory farmed sample, *t*(116) = 2.46, *p* < 0.016, η^2^_p_ = 0.050 (Fig 2) demonstrating that implicit consumption behavior was also influenced by beliefs.

### Discussion

Study 1 supports our hypothesis that beliefs about how animals are raised can influence the experience of eating meat. Participants enjoyed beef jerky less when paired with the factory farm description compared to the humane farm description—even though the meat products were identical. Participants were also willing to pay less and were less likely to eat the factory farmed meat again. Importantly, participants even consumed less when they believed meat came from animals raised on the factory farm suggesting the effect was not due to demand characteristics.

## Study 2

Typically when people make decisions about what to eat, they are not confronted with a description of how meat animals are raised. Therefore in Study 2, we added a control description that did not mention how the animals were raised. This condition also allowed us to test whether the factory farm label reduced enjoyment or humane farm label increased enjoyment. Additionally, Study 2 utilized a between participant design, so each participant sampled only one product. This eliminated the possibility that the effect observed in Study 1 was artificially induced by participants sampling two very similar products.

### Methods

#### Participants

Study 2 took place outdoors on the campus of Northeastern University, Boston, MA, USA. This allowed us to efficiently collect data from a more diverse group of participants [28] and allowed us to test if the effect was robust enough to hold in an uncontrolled context outside the laboratory. Pedestrians (including students, staff, faculty, visitors, and community members) were asked if they would like to participate in a taste study. The initial target sample size from Study 1 was doubled (to n = 300) because the between participant design of Study 2. Data collection was stopped at the end of the semester. 259 people participated in Study 2. Nine people were removed for not following instructions (consuming the sample before reading the description). Two people declined to sample the meat after reading the factory farm description. Therefore the analysis reported here includes 248 people. To keep the study brief, no demographic data was collected, but Northeastern University has a diverse student population: 10% Asian, 3% Black/African-American, 7% Hispanic/Latino, 49% White, and 18% international [29].

#### Materials

Researchers prepared samples of roast beef by placing 2.5 grams of meat in small paper cups with a toothpick. As in the first study, the sample was always the same product, but it was paired with different descriptions to manipulate beliefs. A small table was set up in an area with high foot traffic. Researchers wore red aprons and name tags while collecting data.

A single page of paper was prepared that contained a description of the roast beef (S1 Table) and a scale for participants to indicate how much they liked the roast beef. To manipulate beliefs about the meat, we created four new descriptions (see S1 Table). First, we added a control condition that did not mention animals at all; instead it described where the meat was typically sold. New humane farm and factory farm descriptions were created to be more similar so the only differences were related to animal welfare (organic and antibiotic language used in Study 1 was removed). We also created an additional description of a factory farm that was framed in a relatively positive way (henceforth ‘factory farm+’). The factory farm+ description highlighted one of the advantages of factory farming: it is efficient which allows meat to be affordably produced. This condition was added to test whether highlighting the advantages of factory farming would offset the effects animal suffering.

Participants reported how much they liked the sample using a general Labeled Magnitude Scale anchored by “strongest imaginable dislike” to “strongest imaginable like” [30]. The scale was 100 millimeter long, and participants were instructed to make a mark anywhere along the scale that corresponding to how much they liked the product.

#### Procedure

Researchers invited pedestrians to sample roast beef as part of a brief study. Those who were interested were informed about the study including their rights to refuse to participate and gave verbal consent to continue. Consent was documented by participants’ written responses to experimental questions. No identifying information was collected. Participants were handed a clipboard with a single page that included the description and response scale. Participants were instructed to read the description at the top of the page, then sample the roast beef, and finally, rate how much they liked the meat by marking the visual analogue scale. Because the manipulation in this study was between participants, each participant read only one description and tasted only one sample.

### Results

**Fig 3:**
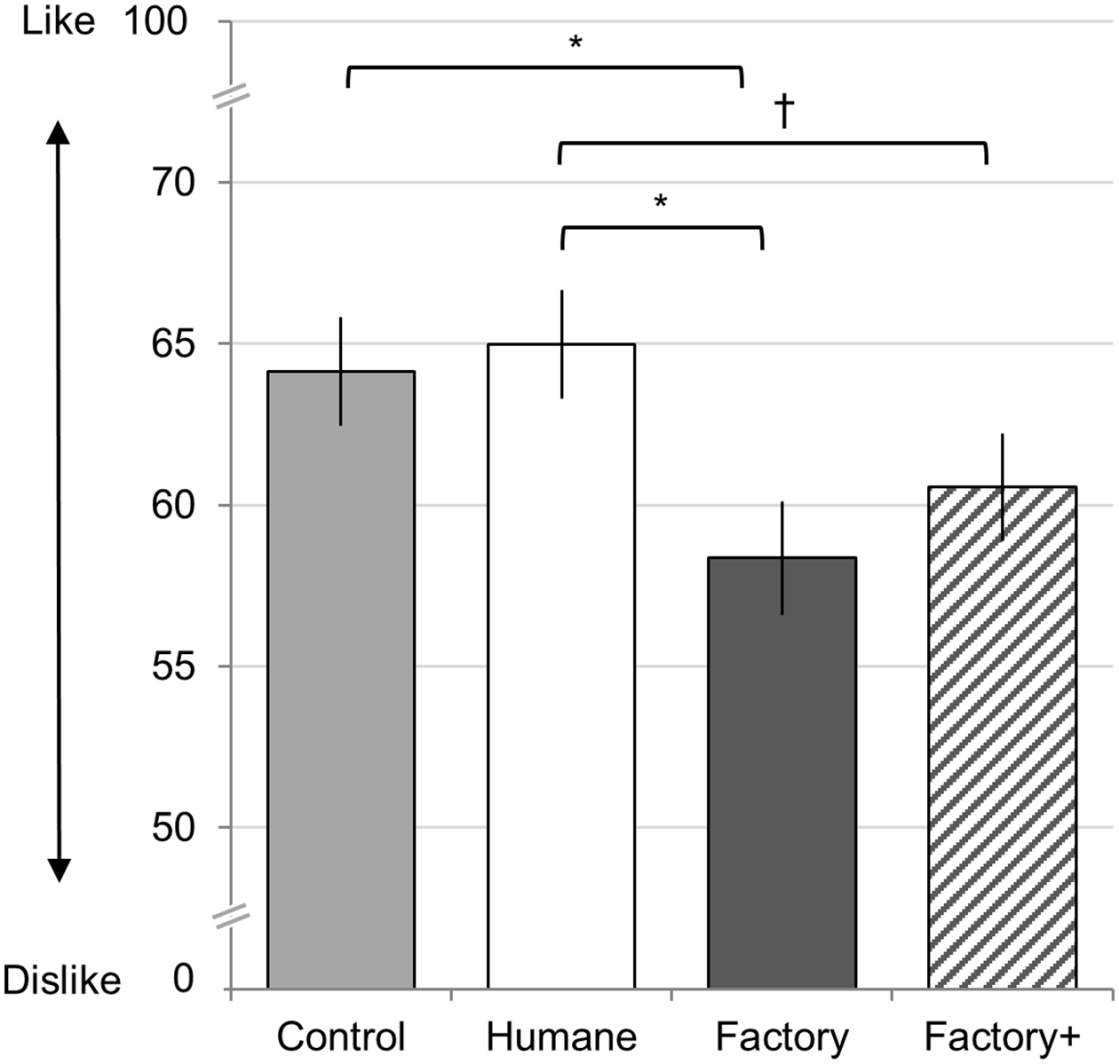
Study 2: Ratings of Roast Beef Sample. Error bars represent standard errors. Asterisks represent significance (* *p* < 0.05), and dagger represents trend († *p* < 0.07). All ratings made on 100 millimeter general Labeled Magnitude Scale.

To test whether beliefs influenced the experience of eating meat, we conducted a one-way ANOVA, with description as the independent variable (control description, humane farm, factory farm, factory farm+) and liking as the dependent variable. Supporting our hypothesis, we found that the descriptions influenced liking, *F*(3, 244) = 3.25, *p* < 0.023, η^2^_p_ = 0.038 (Fig 3; see S2 Table for means, SE, and 95% CI). Follow-up planned independent sample t-tests (two-tailed) revealed that meat paired with the factory farm description was less liked compared to the humane farm, *t*(119) = 2.52, *p* < 0.014, and control description, *t*(119) = 2.33, *p* < 0.022. The humane farm and control condition were approximately equally liked, *t*(124) = 0.37, *p* = .712. Additionally, the factory farm and the positively framed factory farm were approximately equally liked, *t*(120) = 0.85, *p* = 0.398. The difference between the humane farm and positively framed factory farm reached trend level significance, *t*(125) = 1.90, *p* < 0.061.

### Discussion

Study 2 replicated that beliefs about how meat animals were raised can influence the experience of eating. Specifically, knowing that beef was raised in “small cages” (both factory farm conditions) led to lower ratings of liking compared to the humane farm and control descriptions. Interestingly, beef paired with the control description (describing where meat is sold) and the humane farm description were equally liked. That is, the humane farm description does not increase liking, but the factory farm description reduces liking. Meat paired with the factory farm+ description was not more liked than the meat paired with the factory farm, suggesting the effect is not simply driven by one farm being portrayed in a positive manner. Rather, the conditions in which animals are raised is the important factor.

The findings from the first two studies raise an even more compelling possibility: can beliefs influence very basic *sensory* properties of flavor, such as perceived saltiness or sweetness?

## Study 3

To test whether beliefs about how animals were raised can influence basic sensory experience, participants in Study 3 reported on particular properties of flavor (e.g. saltiness, sweetness, etc.). As in the Study 1, participants sampled meat and reported on the experience (e.g. pleasantness, smell, appearance), and implicit eating behavior (amount eaten) was measured.

### Methods

#### Participants

117 Northeastern undergraduate students participated in Study 3 (sample size was determined as in Study 1). Three participants were removed because they did not follow instructions (the pattern of findings do not change with all participants included). Therefore, data were analyzed for 114 participants (55.3% female) from 19 to 24 years old (*M* = 19.12, *SD* = 1.40). Participants were recruited through introductory psychology classes and received credit for participation. All participants gave written, informed consent before participating.

#### Materials

Study 3 used similar materials and study design to Study 1 with the following modifications. First, we created new, more evocative descriptions involving text and pictures of animals (see S1 Table). In the previous study (Study 2), the control condition described where the meat was sold without mentioning animals or farms. Here, in Study 3, we utilized a control condition that had no description of the meat (no text was presented). This mirrors many real world situations in which meat is not paired with any description of how it was produced or where it is sold. Thus, the control condition in Study 3 most closely resembles real world situations in which meat is consumed. In Study 3, the control condition always came first. This was done because once participants read a description, they would be likely to apply it to any subsequent unlabeled samples. Thus, all participants first completed the control condition, and then the other two conditions (order of the final two conditions was randomized).

In Study 3, we used samples of deli ham that were prepared by cutting thickly sliced ham into five pieces weighing a total of five grams. Three separate samples were prepared for each participant, and each was placed into a paper sample cup and was lightly heated in a microwave oven for 5 seconds. Samples were kept warm by placing them in an insulated box to retain heat until consumed in the experiment.

#### Procedure

The procedure in Study 3 was similar to Study 1. Participants tasted three samples. Participants always tasted the ham paired with no description first. Next, participants sampled ham paired with either the factory farm description or the humane farm description (order randomized) and then sampled ham paired with the remaining description.

Each trial started with participants cleansing their palate by drinking a small amount of water. Next participants read the farm description (S1 Table), consumed the sample, and reported their experience of the sample. First, participants rated how pleasant the overall consumption experience was on a 100-point slider scale (from 0 = ‘very unpleasant’ to 100 = ‘very pleasant’). Next participants rated different taste properties of the sample: savory, salty, sweet, bitter, sour, fresh, and greasy (from 0 = ‘not very much’ to 100 = ‘very much’). Next participants rated how pleasant the sample tasted, appeared, and smelled (from 0 = ‘very unpleasant’ to 100 = ‘very pleasant’). Next, participants reported how much they would be willing to pay for 16 ounces of the sample (from $0 to $20) and how likely they would be to eat the sample again (from 0 = ‘would never eat this again’ to 100 = ‘would definitely eat this again’). As in Study 1, we measured how much of the sample was consumed by measuring the weight of the samples before and after participants completed the taste test. Participants were free to eat as much of the samples as they wanted, so the amount consumed could vary from zero to five grams.

To check whether the manipulation materials (the descriptions) were perceived as negative or positive, participants were asked to rate the descriptions at the end of the experiment. Participants were asked how pleasant the descriptions were (from 0 = ‘very unpleasant’ to 100 = ‘very pleasant').

To check whether participants were attending to the farm descriptions, participants were asked to recall the descriptions after they completed the sampling procedure. Only three participants did not recall any parts of the description suggesting they did not read them. These participants were removed from analysis, but including them does not change the pattern of findings.

### Results

**Fig 4:**
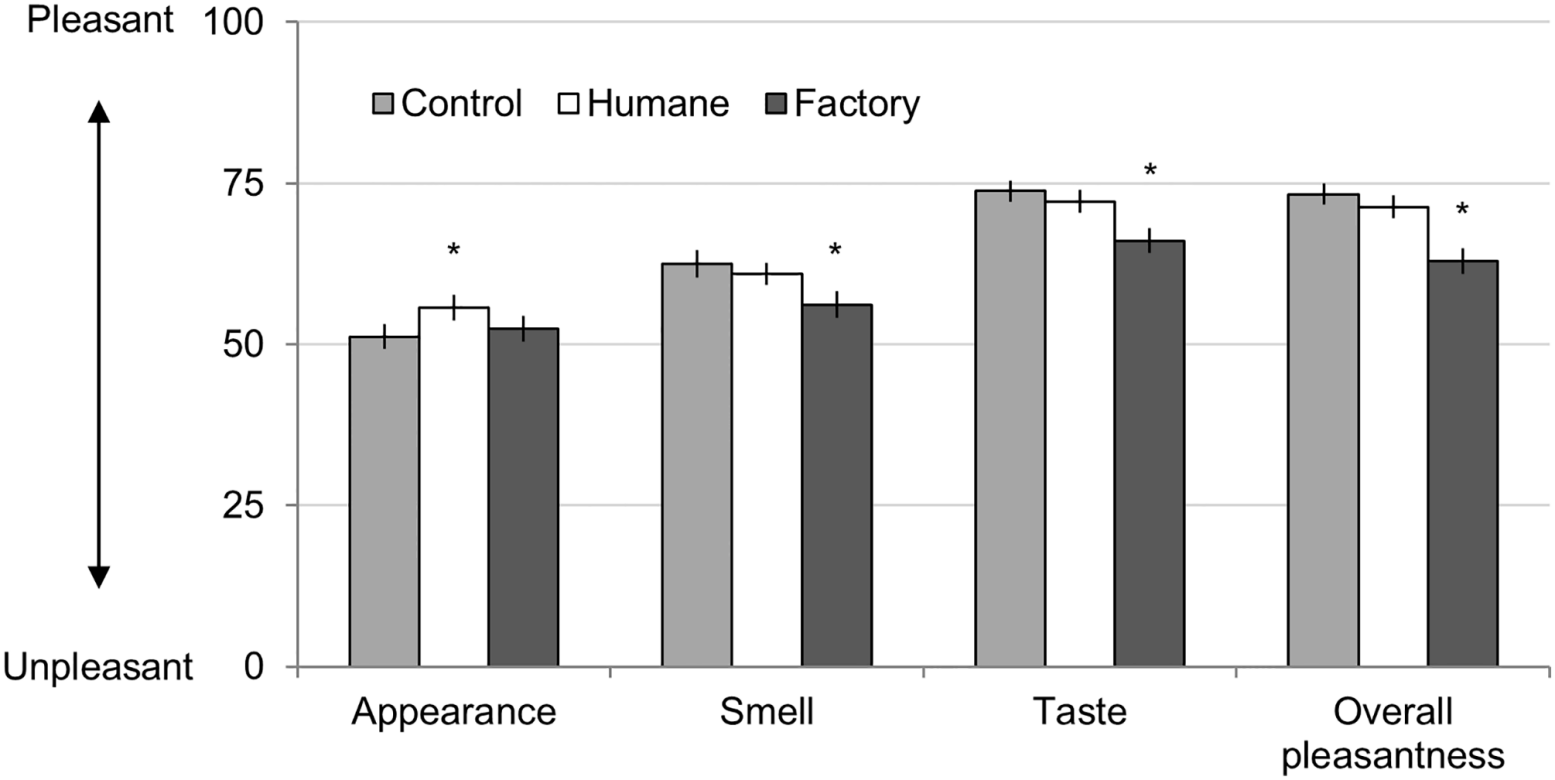
Study 3: Ratings of Ham Sample. Error bars represent standard errors, and asterisks represent significance (* *p* < 0.05). All ratings made on 100-point slider scales.

**Fig 5:**
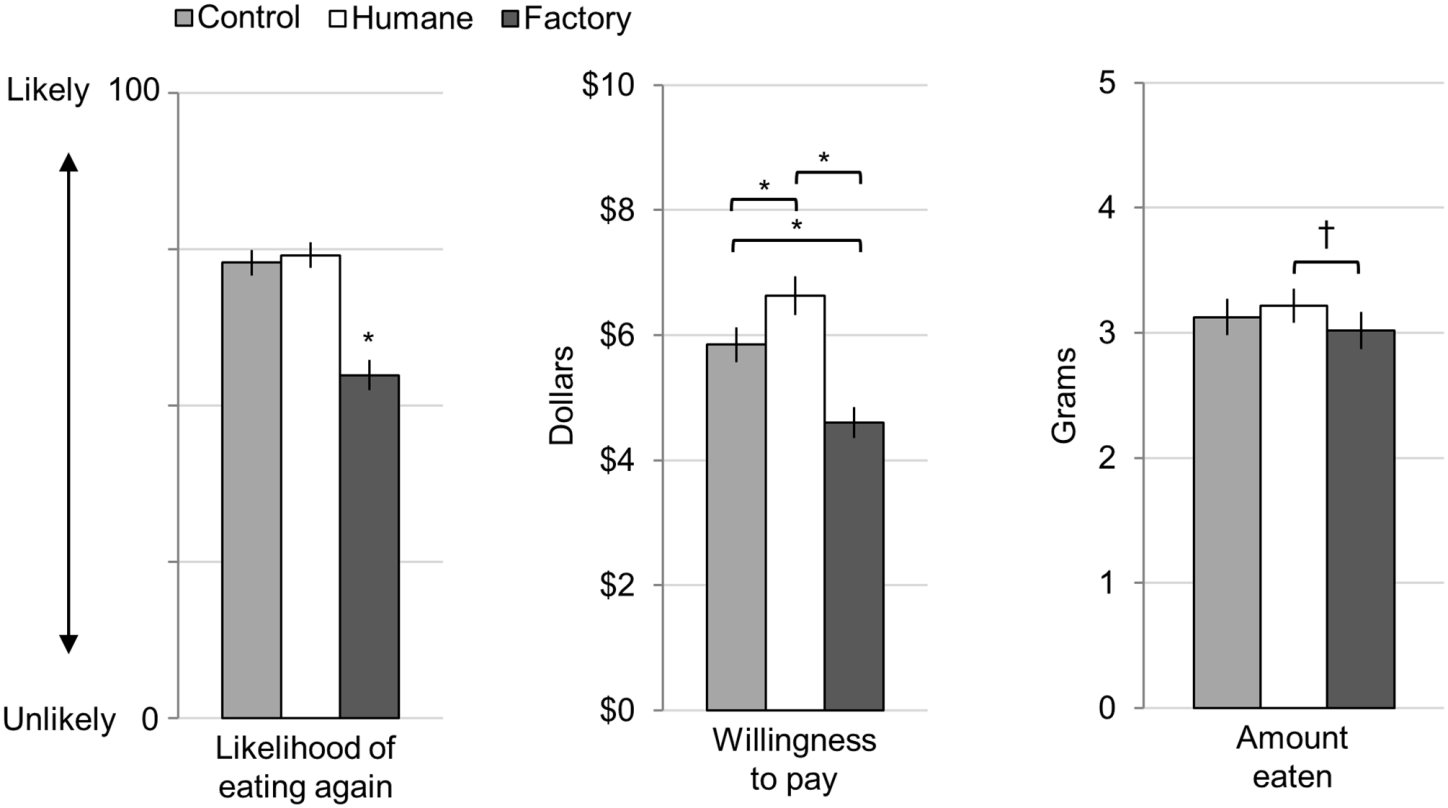
Study 3: Behavioral Measures for Ham Sample. Error bars represent standard errors. Asterisks represent significance (* *p* < 0.05), and dagger represents trend († *p* < 0.07). All ratings were made on 100-point slider scales.

**Fig 6:**
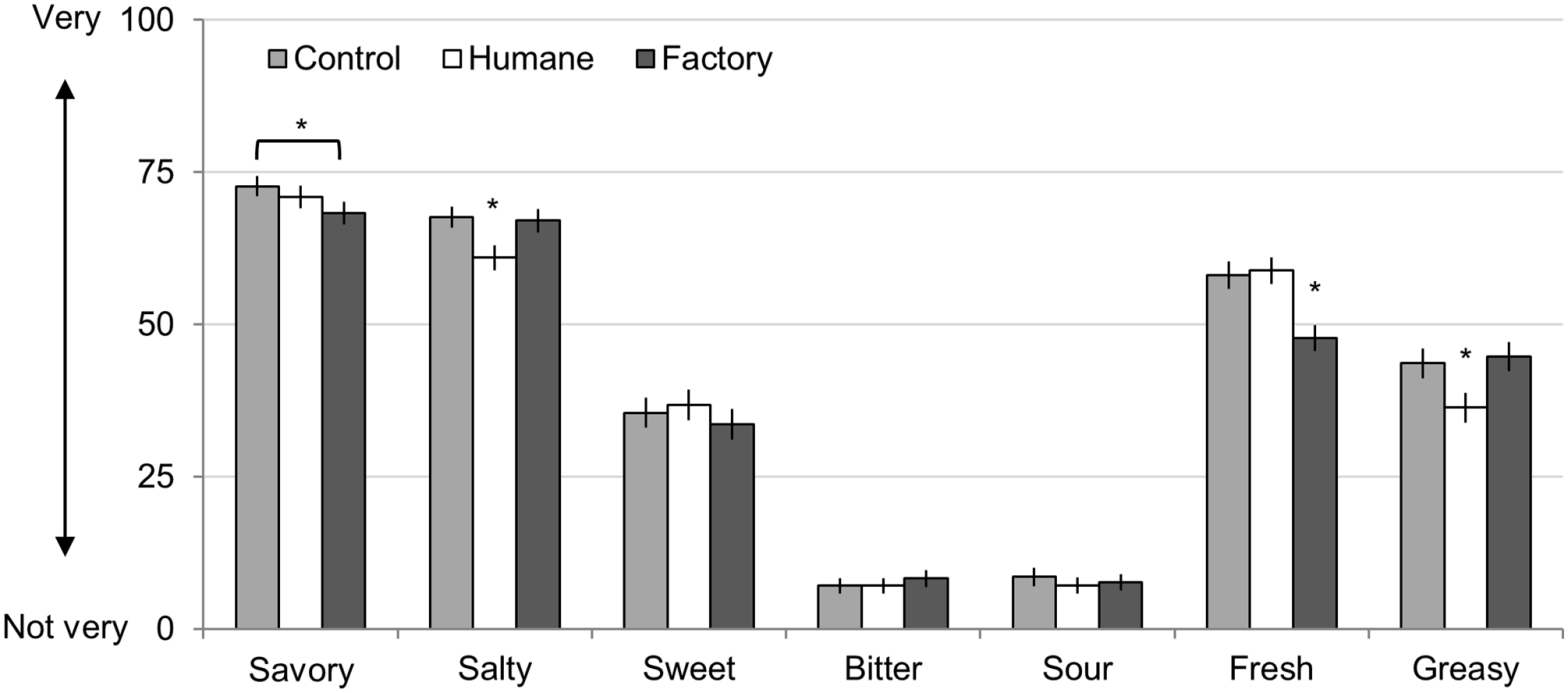
Study 3: Flavor Ratings of Ham Sample. Error bars represent standard errors, and asterisks represent significance (* *p* < 0.05). All ratings made on 100-point slider scales.

To test whether beliefs influenced the eating experience, we conducted a MANOVA with description (humane farm, factory farm, control) as a repeated measure and participants’ ratings as the dependent variables. The MANOVA was significant, Wilks' Lambda = 0.42, *F*(28,282) = 5.49, *p* < 0.001, η^2^_p_ = 0.353. To understand this effect, we ran a series of univariate tests (a one-way repeated measure ANOVA for each dependent variable). Greenhouse—Geisser corrected tests are presented if the sphericity assumption was not met (S2 Table reports complete statistics and which tests are corrected). Supporting our hypothesis, we found the descriptions influenced the pleasantness of taste, appearance, smell, and overall experience (all *F*s > 3.51, all *p*s < .05; see S2 Table for complete statistics). To understand which of the three conditions differed, we visually inspected the means (Figs 4–6) and computed least significance difference tests (LSD; S2 Table reports LSD post-hoc results, means, SEs, and 95% confidence intervals). Post-hoc tests revealed that the factory farmed sample was rated as significantly less pleasant compared to the humane sample. In general, the control sample was rated similarly to the humane sample (replicating our findings in Study 2). The one exception is that the *appearance* of the control sample was rated as pleasant as the factory farm sample (see Fig 4; see S2 Table for complete statistics).

The descriptions also influenced participants' self-reported intentions (Fig 5; see S2 Table for complete statistics). Descriptions influences participants' reports of how likely they were to eat the meat again, *F*(1.70,188.41) = 53.07, *p* < 0.001, η^2^_p_ = 0.32. In particular, post-hoc tests show participants reported being less likely to consume the factory farmed sample again compared to the other two samples. Descriptions also influenced how much participants were willing to pay for 16 ounces of the sample, *F*(1.63,180.74) = 41.74, *p* < 0.001, η^2^_p_ = 0.027. Post-hoc tests revealed participants were willing to pay significantly different amounts for each sample: they were willing to pay the most for meat paired with the humane description ($6.63), followed by the control description ($5.85), and least for the factory farm description ($4.61).

Additionally, the descriptions influenced participants' implicit behavior. Descriptions influenced the amount of the sample consumed at a trend level of significance, *F*(2,224) = 2.99, *p* = 0.052, η^2^_p_ = 0.026. This difference was driven by participants consuming 6.19% less of the factory farm sample compared to the humane sample (Fig 5).

Finally, descriptions also influenced basic properties of flavor (Fig 6; see S2 Table for complete statistics). Specifically, the descriptions influenced ratings of how savory, salty, fresh, and greasy the samples were (all *F*s > 3.94, all *p*s < 0.022). The descriptions did not significantly influence ratings of how sweet, bitter, or sour the samples were (all *F*s < 2.02, all *p*s > 0.134). Post-hoc tests show that meat paired with the humane sample was experienced as less salty and greasy than the other two samples. Meat paired with the factory farmed sample was experienced as less fresh than the other two samples. The meat paired with the control description was experienced as more savory than the meat paired with the factory farm description (meat paired with the humane description was intermediately savory).

The manipulation check confirmed that participants reported the descriptions significantly differed in terms of pleasantness, *F*(1.50,108.08) = 59.17, *p* < 0.002, η^2^_p_ = 0.45. Post-hoc tests confirmed that participants rated the factory farm description as significantly less pleasant compared to the control condition, and the humane farm was significantly more pleasant compared to the control condition (see S2 Table).

### Discussion

Study 3 again found that beliefs about animal welfare influenced the experience of eating. Replicating Study 1, participants reported meat paired with the factory farm description was less pleasant in terms of smell, taste, and overall experience (compared to meat paired with the humane farm description). Participants reported being less likely to eat the factory farmed sample again, and were willing to pay less for it. Additionally, they actually ate less of the factory farmed sample. Replicating Study 2, the control condition was similar to the humane farm condition. Intriguingly, Study 3 also found that beliefs influence the basic sensory properties of flavor: participants reported the factory farmed meat was less fresh, more salty, and more greasy (compared to meat paired with the humane farm description).

## General Discussion

What's for dinner matters. The decision to eat *meat* is particularly important because eating too much can increase the prevalence of metabolic related diseases, including cancer, heart disease, and obesity [31]. Eating too much red meat, in particular, also increases mortality rates [32]. Consumer demand for meat supports industrialized animal farming, which some argue causes mass animal suffering [15]. While health and ethical considerations lead some people to avoid meat [17], many still choose to eat meat—largely because eating meat is hedonically pleasant [33]. Ethical production of goods is becoming increasingly important in purchasing decisions (for discussion see [12]). Emerging research has explored whether beliefs about food production influence consumption experiences [11–13,34], but no work has focused on meat. We hypothesized that beliefs about how farm animals are raised would shape the experience of eating meat.

Supporting our hypothesis, three studies found that beliefs about how animals were raised influenced the experience of consuming meat. In particular, factory farmed meat looked, smelled, and tasted less pleasant. People were willing to pay less and reported being less likely to eat factory farmed meat again. Additionally, the perceived flavor of meat was also influenced by beliefs: factory farmed meat tasted more salty, more greasy, and less fresh compared to humanely raised meat. Interestingly, perceptions of how sweet, bitter, and sour were not influenced. While we do not know why only some properties were influenced, one possibility is that people expect factory farmed meat to be more salty (but not more bitter), and these expectations drive the changes in perception [9]. Finally, people actually consumed less meat when they believed it came from a factory farm compared to a humane farm. Though the difference was small, the reduction demonstrates that beliefs can influence actual eating behavior.

While negative beliefs (factory farms) reduced enjoyment, positive beliefs (humane farms) did not increase enjoyment. That is, believing meat came from a humane farm did not boost the overall hedonic experience compared to meat paired with a control description (Study 2) or no description (Study 3). This pattern is consistent with other studies that find negative information is more impactful than positive information. For instance, negative (but not positive) social information (gossip) influences visual processing of faces [35]. Additionally, negative environmental labeling seems to drive consumer preference more than positive labeling [36]. This weighting of negative information is consistent with a negativity bias (for review see [37]) and loss aversion [38]. One reason negative information might be so powerful is that the cost of missing a negative stimulus could result in catastrophic loss while missing a positive stimulus only results in missing a slightly positive gain. For instance, eating a poisonous food could result in death, while missing an edible food only results in reduced caloric consumption. A second possibility is that people might have idealized pastoral beliefs about how animals are typically raised (i.e. contented cows lounging in lush green pastures). If these are people’s typical beliefs, the humane and control conditions in our studies might have led to generally the same beliefs in participants (i.e. that animals were raised in humane conditions). A third alternative possibility is that people do not typically think about where meat comes from, and any reminder that meat comes from animals is unpleasant [18,19,39]. That is, when giving information about how farm animals are raised, there is simply no getting around the fact that meat is animal flesh and this interferes with people's ability to enjoy meat. Our finding that negative, but not positive, beliefs influence the experience of eating stands in contrast to other studies that found ‘fair-trade’ labels seem to boost the pleasantness of chocolate [12,40], and ‘local’ labels seem to boost the pleasantness of juice [12]. While more research is clearly needed, one possibility is that there is something special about meat and beliefs about animal suffering. For instance, affective responses to animal suffering are likely to be more powerful than affective responses to non-organic, non-fair-trade, or non-local food production. These varied findings also point out the need to understand what beliefs people typically have about food (before entering laboratories) and highlight the need to be particularly careful about how those beliefs are made salient through study instructions and study designs. The finding that negative labels have an effect, but positive ones do not seem to, could prove to be a challenge for producers who advertise and sell ethically produced products. The present findings suggest that reporting humane treatment is no better than saying nothing when it comes to how the food is experienced. Positive beliefs generally did not boost how positively the meat was experienced or how much was eaten. Raising animals humanely is more expensive (and thus the product is more expensive), so producers might need to find another way to attract consumers. This concern may be offset by our finding that people are willing to pay more for humanely raised meat. Why people report being willing to will pay more for a humane product that is not more pleasant is an open question.

The three studies reported here demonstrate the phenomenon that beliefs about how animals are raised influence the experience of eating meat; future work will be required to unpack the mechanism driving the effect. One possibility is that affective beliefs may influence experience through affective realism [41]. According to affective realism, affect is a fundamental property of conscious experience [42,43], so people experience the world as intrinsically affective: paintings are beautiful or ugly; food is delicious or disgusting. According to affective realism, beliefs in the current studies are conceptual knowledge represented in a distributed, multi-modal fashion that include affective and sensory representations [22]. The belief that meat was raised on a factory farm has potency because the belief is affective and includes an embodied representation of animal suffering (mirroring; for review see [23]). According to affective realism, these embodied affective beliefs set the neural context for incoming sensory information, which is then integrated with those existing representations to construct a unified, conscious eating experience. While supported by existing theory, this possible mechanism needs to be tested with future research.

### Weaknesses

Our studies primarily rely on self-reported experience, which can be influenced by demand characteristics. Perhaps in Studies 1 and 3, participants experienced all of the products in the same way, but felt pressure to change their explicit ratings to be more desirable. There are four ways we attempted to address this possibility. First, experimenters were always blind to the condition while participants tasted and rated the product (except the control condition in Study 3). This was done so the experimenters could not influence responses by unknowingly changing their behavior. Second, participants were always instructed that there was no correct response and that their responses would not be linked to their names. Third, in Studies 1 and 3 responses were made on a computer so the researcher was unaware of participants’ responses to reduce pressure participants may have felt. Finally, and most important, we measured liking implicitly by measuring the amount of meat consumed (Studies 1 & 3). We found that participants consumed less meat when they believed it was raised on a factory farm, suggesting they enjoyed it less. It seems unlikely that participants would carefully control the amount of the samples they consumed for social desirability. However, future research could use additional implicit or physiological measures to rule out the possibility that the effects observed are not driven by demand characteristics.

### Conclusion

We found that affective beliefs about animal welfare influence the experience of eating meat, but this effect likely extends to any strong affective belief (such as other moral violations like purity or hierarchy norms; see [44]. Additionally, these effects almost certainly extend beyond meat to other food, and even other experiences: knowing that an artist committed a crime might change the experience of their art. These findings suggest that anyone interested in creating an experience (e.g. film-makers, designers, chefs) should consider how beliefs influence the user experience. Broadly, this work suggests top-down influences (such as affective beliefs) play an important role in shaping experience. Experience is not determined solely by physical properties of the external world—experience is also shaped by beliefs.

## Supporting Information

S1 Table Description of Meat Samples. (PDF)

S2 Table Study 1–3 Means, Standard Errors, 95% Confidence Intervals, and Test Statistics. Each line represents a separate omnibus ANOVA test. Follow-up post-hoc comparisons were done using Least Significant Difference procedure and are reported with letters (a, b, c). Means with different letters are significantly different. * denotes omnibus ANOVA tests adjusted with Greenhouse-Geisser correction.
(PDF)
