# Supplementary material for: Affective Beliefs Influence the Experience of Eating Meat
Source: PLoS One. 2016 Aug 24;11(8):e0160424. doi: 10.1371/journal.pone.0160424 (PMC4996498; doi:10.1371/journal.pone.0160424)
Supplement: S1 Table — (PDF) [file pone.0160424.s001.pdf]

	Control	Humane Farm	Factory Farm	Factory Farm+
Study 1 (jerky)		This humanely raised beef jerky was raised on a family farm. The animals grazed in outdoor pastures and consumed organic feeds. They were not raised with antibiotics or artificial growth hormones. Care was taken to ensure the welfare of the animals. This product meets all USDA organic certification standards.	This beef jerky was produced on a factory farm. The animals were confined in pens where they were unable to lay down. They were given antibiotics and hormones to speed growth. No steps were taken to ensure the welfare of the animals. This product meets all USDA certification standards.	
Study 2 (roast beef)	This beef comes from Hillman's, a store that sells sliced deli meat. This product can be used in a variety of meals including sandwiches. Hillman's products pass all federal requirements and are sold around the country.	This beef was raised at Hillman's, a farm that values animal welfare. To more humanely raise animals, their cows roam in grassy outdoor pastures. Hillman's is recognized with awards for their exceptional treatment of farm animals.	This beef was raised at Hillman's, a facility that values production. To more easily produce meat, their cows are kept in small indoor cages. Hillman's products pass all federal requirements and are sold around the country.	This beef was raised at Hillman's, a facility that values efficiency. To more affordably provide meat, their cows are kept in small indoor cages. Hillman's products pass all federal requirements and are affordably priced for everyone.
Study 3 (ham)	(no description)	This ham was raised on a farm that focused on animal welfare. The animals were allowed to roam in grassy outdoor pastures where they could graze and exercise. The animals were not isolated so were able to engage in social behaviors with other pigs.	This ham was produced at a factory farm that focused on production. The animals were confined to concrete indoor pens where they were unable to lie down or go outside. The animals were isolated so could not engage in social behaviors with other pigs.	
		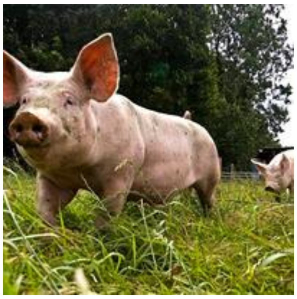	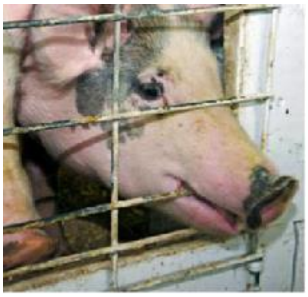	
