# Supplementary material for: Affective Beliefs Influence the Experience of Eating Meat
Source: PLoS One. 2016 Aug 24;11(8):e0160424. doi: 10.1371/journal.pone.0160424 (PMC4996498; doi:10.1371/journal.pone.0160424)
Supplement: S2 Table — Each line represents a separate omnibus ANOVA test. Follow-up post-hoc comparisons were done using Least Significant Difference procedure and are reported with letters (a, b, c). Means with different letters are significantly different. * denotes omnibus ANOVA tests adjusted with Greenhouse-Geisser correction. (PDF) [file pone.0160424.s002.pdf]

	Control					Humane Farm					Factory Farm					Factory Farm+					Omnibus test			Greenhouse-Geisser correction
	Mean	SE	95% CI Lower	95% CI Upper	LSD	Mean	SE	95% CI Lower	95% CI Upper	LSD	Mean	SE	95% CI Lower	95% CI Upper	LSD	Mean	SE	95% CI Lower	95% CI Upper	LSD	*F*	*p*	η^2_p	
Study 1 (jerky)																								
Appearance						40.76	2.14	36.53	44.99		33.57	2.08	29.45	37.68							25.88	0.001	0.19	
Smell						53.95	2.46	49.08	58.82		50.93	2.32	46.33	55.53							6.10	0.015	0.05	
Taste						55.95	2.45	51.09	60.80		50.82	2.48	45.91	55.73							9.61	0.002	0.08	
Overall enjoyment						56.09	2.53	51.08	61.09		49.02	2.48	44.10	53.93							17.41	0.001	0.13	
Willingness to pay						3.64	0.21	3.23	4.05		2.85	0.19	2.48	3.22							44.03	0.001	0.28	
Likelihood of eating again						59.32	3.00	53.36	65.27		48.01	3.09	41.90	54.12							34.33	0.001	0.24	
Amount eaten						0.77	0.06	0.67	0.89		0.71	0.06	0.60	0.82							6.05	0.015	0.05	
Study 2 (roast beef)																								
Overall liking	64.14	1.69	60.82	67.46	a	64.97	1.69	61.65	68.29	a	58.36	1.76	54.90	61.82	b	60.55	1.67	57.25	63.84	ab	3.25	0.022	0.04	
Study 3 (ham)																								
Appearance	51.19	1.95	47.33	55.05	a	55.67	1.93	51.84	59.51	b	52.38	2.00	48.40	56.35	a						3.51	0.036	0.03	*
Smell	62.51	2.11	58.32	66.70	a	60.93	1.74	57.48	64.38	a	56.14	2.09	51.99	60.29	b						7.37	0.001	0.07	
Taste	73.79	1.66	70.51	77.08	a	72.14	1.79	68.60	75.68	a	66.09	1.88	62.36	69.82	b						15.67	0.001	0.13	
Overall pleasantness	73.30	1.66	70.01	76.59	a	71.31	1.81	67.73	74.89	a	62.93	1.96	59.05	66.80	b						20.83	0.001	0.16	
Willingness to pay	5.85	0.27	5.30	6.39	a	6.63	0.31	6.02	7.24	b	4.61	0.25	4.12	5.10	c						41.74	0.001	0.27	*
Likelihood of eating again	72.88	2.06	68.80	76.95	a	74.06	2.00	70.11	78.02	a	54.86	2.42	50.07	59.65	b						53.07	0.001	0.32	*
Amount eaten	3.13	0.15	2.84	3.41	a	3.22	0.14	2.95	3.48	b	3.02	0.15	2.72	3.32	a						2.99	0.052	0.03	
Savoury	72.72	1.71	69.34	76.10	a	70.91	1.88	67.18	74.64	ab	68.28	1.91	64.50	72.05	b						3.94	0.021	0.04	*
Salty	67.67	1.74	64.22	71.12	a	60.94	2.09	56.79	65.08	b	67.05	1.90	63.28	70.81	a						13.79	0.001	0.11	*
Sweet	35.52	2.44	30.69	40.35		36.80	2.55	31.76	41.85		33.59	2.55	28.54	38.64							2.02	0.135	0.02	*
Bitter	7.11	1.26	4.61	9.61		7.10	1.26	4.60	9.60		8.30	1.38	5.56	11.03							0.86	0.423	0.01	*
Sour	8.53	1.53	5.50	11.55		7.16	1.32	4.54	9.78		7.63	1.35	4.96	10.29							1.12	0.328	0.01	*
Fresh	58.12	2.22	53.72	62.52	a	58.83	2.18	54.50	63.16	a	47.71	2.11	43.53	51.89	b						23.78	0.001	0.18	
Greasy	43.64	2.45	38.78	48.50	a	36.33	2.44	31.49	41.18	b	44.77	2.39	40.04	49.50	a						16.15	0.001	0.13	
Description pleasantness (manipulation check)	60.66	2.46	55.76	65.56	a	77.81	1.99	73.84	81.78	b	39.49	3.79	31.94	47.05	c						59.17	0.001	0.45	*
